# Supplementary material for: Sustained-input switches for transcription factors and microRNAs are central building blocks of eukaryotic gene circuits
Source: Genome Biol. 2013 Aug 23;14(8):R85. doi: 10.1186/gb-2013-14-8-r85 (PMC4054853; doi:10.1186/gb-2013-14-8-r85)
Supplement: Additional file 5 — HTML Browsable Motif Output. Zipped folder containing all WaRSwap and FANMOD motif output, viewable in a web browser. [file gb-2013-14-8-r85-S5.ZIP › HTML_browsable_motif_output/WaRSwap_ath_tair9/sigs_WSWRSLDraw_2-2000.pvals.heatmaps.html/motif_id_38_000101101_tftype_ath_upstream_-2000_0.html]

```
BG_MODEL = Rnew2
MOTIF_ID = 38_000101101
TF_TYPE = ath
UPSTREAM = -2000_0


PVals
FN_0.2	FN_0.4	FN_0.6	FN_0.8
dg_60.genes	0.149	0.377	0.034	0.004
dg_70.genes	0.244	0.363	0.024	0.004
dg_80.genes	0.091	0.183	0.013	0.006

ZScores
FN_0.2	FN_0.4	FN_0.6	FN_0.8
dg_60.genes	1.076	0.268	2.046	4.043
dg_70.genes	0.76	0.355	2.228	4.095
dg_80.genes	1.402	0.956	2.728	4.247

StDevs
FN_0.2	FN_0.4	FN_0.6	FN_0.8
dg_60.genes	57.998	30.222	10.55	3.235
dg_70.genes	57.512	28.888	10.031	3.035
dg_80.genes	52.956	26.724	9.444	3.016
```
